# Cross-reactivity IgG, viral load, severity and vaccination outcome as an approach for understanding humoral immune response against SARS-CoV-2

**DOI:** 10.1186/s12879-025-12038-3

**Published:** 2025-11-18

**Authors:** Jesus Contreras-Villa, Griselda Rodríguez-Martínez, Israel Parra-Ortega, Mariana Romo-Castillo, Karen Cortés-Sarabia, Zeus Saldaña-Ahuactzi, Alejandro Flores-Alanis, Alfredo Aureoles-Romero, Marcela Salazar-García, James González, Carlos A. Eslava-Campos, Ulises Hernández-Chiñas, Armando Cruz-Rangel, Rosario Morales-Espinosa, Mario Eugenio Cancino-Diaz, Victor M. Luna-Pineda

**Affiliations:** 1 Laboratorio de Investigación en Patógenos Respiratorios, Hospital Infantil de México Federico Gómez, Mexico City, Mexico; 2 Departamento de Inmunología, Escuela Nacional de Ciencias Biológicas, Instituto Politécnico Nacional, Mexico City, Mexico; 3 Departamento de Laboratorio Clínico, Hospital Infantil de México Federico Gómez, Mexico City, Mexico; 4 IxM, Consejo Nacional de Ciencia, Humanidades y Tecnología/Hospital Infantil de México Federico Gómez, Mexico City, Mexico; 5 Laboratorio de Inmunobiológica y Diagnóstico Molecular, Facultad de Ciencias Químico-Biológicas, Universidad Autónoma de Guerrero, Chilpancingo de los Bravo, Guerrero Mexico; 6 Centro de Investigación en Biotecnología Aplicada, Instituto Politécnico Nacional, Santa Inés Tecuexcomac, Tepetitla de Lardizábal, Tlaxcala Mexico; 7 Departamento de Microbiología y Parasitología, Facultad de Medicina, Universidad Nacional Autónoma de México, Mexico City, Mexico; 8 Laboratorio de Biología del Desarrollo y Teratogénesis Experimental, Hospital Infantil de México Federico Gómez, Mexico City, Mexico; 9 Laboratorio de Biología Molecular y Genómica, Departamento de Biología Celular, Facultad de Ciencias, Universidad Nacional Autónoma de México, Mexico City, Mexico; 10 Laboratorio de Patogenicidad Bacteriana, Facultad de Medicina-UNAM, Hospital Infantil de México Federico Gómez, Mexico City, Mexico; 11 Laboratorio de Bioquímica de Enfermedades Crónicas, Instituto Nacional de Medicina Genómica, Mexico City, Mexico

**Keywords:** Antibodies, Humoral immune response, SARS-CoV-2, Coronaviruses, Spike, Nucleocapsid, COVID-19, Vaccine

## Abstract

**Background:**

Serological evaluation plays a crucial role in understanding cross-reactivity, the prevalence of infection, immune response in COVID-19 disease, asymptomatic infections, and vaccine effectiveness.

**Methods:**

Recombinant spike (rS) and Nucleocapsid (rN) proteins from SARS-CoV-2 were used to determine IgG antibodies (Abs) in serum samples obtained from Mexican adults and paediatrics before and during the pandemic by enzyme-linked immunosorbent assay.

**Results:**

Human sera from 2003 to 2016 showed higher levels of cross seropositivity (54.5‒75%) against rS and rN. In serum samples from adult patients with COVID-19, the reactivity intensity (RI) depended on the severity of the disease, whereas in convalescent paediatric patients with COVID-19, SARS-CoV-2 viral load depended on sex and comorbidities. Regarding vaccine effectiveness monitoring, an increased RI of anti-rS IgG was observed in people vaccinated against COVID-19 who had a natural infection with SARS-CoV-2. During the vaccination scheme, an increase in IgG Abs level was observed with the second dose, whereas a decrease was observed after six months of vaccination. Vaccine boosters increased RI in either homologous/heterologous administration of mRNA and non-replicating viral vector vaccines.

**Conclusions:**

Epidemiological outbreaks and the circulation of non-SARS-CoV-2 coronaviruses may contribute to the primary causes of the observed cross-reactions in antibodies. Furthermore, factors such as viral load and disease severity in infected patients, prior illnesses, the dosage of vaccine and booster shots, and the type of vaccine used in COVID-19-vaccinated individuals may also influence the increase in IgG antibodies. Assessing the antibody-based humoral immune response in serum samples collected before and during an outbreak or pandemic could aid in comprehending emerging and re-emerging diseases and developing effective preventive strategies.

**Clinical trial number:**

Not applicable.

**Supplementary Information:**

The online version contains supplementary material available at 10.1186/s12879-025-12038-3.

## Background

Coronaviruses (CoVs) are members of the family *Coronaviridae*, the subfamily *Coronavirinae* and the order *Nidovirales*. They are subdivided into four genera, the α, β, γ, and δ, but only α and β infect mammals, including humans [1]. CoVs are enveloped viruses with a single-stranded and positive-sense RNA genome of approximately 30 kilobases (kb) [2]. The CoV genome is divided into the ~ 20 kb region that encodes the non-structural proteins and the ~ 10 kb region that encodes the structural and accessory proteins, which possess a high identity among CoVs [3].

Human (h) CoVs are known for causing respiratory diseases ranging from mild to severe. The hCoV-229E, hCoV-OC43, hCoV-NL63, and hCoV-KU1 are the less virulent hCoVs causing the common cold [4]. Regarding severe acute respiratory syndrome (SARS)-causing hCoVs, SARS-CoV-1 caused the first outbreak in Guangdong Province in China, spreading to 26 countries worldwide [5]. Nine years later, the Middle East respiratory syndrome (MERS)-CoV, a novel hCoV epidemic, affected Saudi Arabia, America, Europe, and Africa [6]. In 2019, SARS-CoV-2 was first reported in Wuhan, Hubei Province, China, responsible for the COVID-19 pandemic that has produced >6 million deaths [7].

To overcome the severe diseases caused by these hCoVs, vaccine research was developed using the main structural proteins, such as envelope glycoproteins spike (S) and nucleocapsid (N) [8, 9]. The S protein recognises the angiotensin-converting enzyme and the transmembrane serine protease type III to allow the virus to bind and fuse with the host membrane [10, 11]. Following viral fusion, the N protein enters the host cell with the viral RNA to facilitate viral particle replication, assembly and release [12]. Since these proteins are abundantly expressed during infection and show high immunogenicity, they are good antigen candidates [13].

The vaccine against SARS-CoV-2 was the first approved hCoV vaccine by health regulatory authorities in several countries [14]. Vaccines based on lipid nanoparticle-encapsulated nucleoside-modified messenger RNA (mRNA-1273 and BNT162b2) and non-replicating viral vectors (Adv5-nCoV, ChAdOx1 nCoV-19, and Sputnik V), both encoding the full-length S protein from SARS-CoV-2, were administered to the Mexican population to combat the COVID-19 pandemic in homologous and heterologous prime-boost vaccination strategies (https://vacunacovid.gob.mx/calendario-vacunacion/). These COVID-19 vaccines showed an effectiveness of ~ 90% and induced neutralising (n) antibodies (Abs) production against the S protein to block virus invasion. The emergence of variants of concern (VOC) of SARS-CoV-2 (alpha to omicron) carrying some mutations in the S protein and leading to enhanced transmission and infectivity, increased severity of disease, and decreased production of nAbs, generated worry about vaccine effectiveness against the new VOC [15]. However, observational studies demonstrated that vaccine effectiveness against VOC only decreased from 55.9% (omicron VOC) to 88% (alpha VOC) [16].

SARS-CoV-2 infection mainly remains asymptomatic in children, whereas most older adults have moderate to severe COVID-19 [17]. This differential COVID-19 symptomatology among the population is mainly determined by the viral load of SARS-CoV-2, which modulates the immune response. Nevertheless, as the S and N proteins share 50 to 90% identity among hCoVs, SARS-CoV-2’s cross-reactivity with other hCoVs is likely. Despite nAbs against SARS-Cov-2 having been reported in several cross-reactivity studies, Guo et al. (2021) showed that anti-S HCoV-OC43 Abs were higher in severe cases, especially in Chinese patients who required mechanical ventilation [18]. The S2 domain from S protein is a common epitope between SARS-CoV-2 and hCoV-OC43, so that it could account for immune complex formation (Fcγ activation) [19–21]. Because of such contrasting data, it is imperative to better understand COVID-19, asymptomatic carriers, and vaccine effectiveness in every country. Therefore, in this work, IgG Abs were evaluated in serum samples obtained before and during the pandemic in the Mexican population.

## Materials and methods

### Serum samples collection

The sample collection times and cohorts used in this study are described below. Supplementary Fig. 1 gives a graphical representation of the sera collection times in each cohort. Sera were obtained from whole blood by centrifugation and stored at -80 °C until use.

The Department of Central Laboratory from Hospital Infantil de México Federico Gómez (HIMFG) preserves representative serum samples in a biobank as part of the laboratory’s good quality practices and guidelines. Two hundred and twenty serum samples from the biobank, including those from 2003 (*n* = 44), 2009 (*n* = 44), 2011 (*n* = 44), 2016 (*n* = 44), and 2017 (*n* = 44), were used to determine cross-reactive Abs. The original experimental design considered seasonality when selecting pre-pandemic serum samples. However, as no epidemiological data for hCoVs existed before the COVID-19 pandemic in Mexico, the years used in this study were based solely on sample availability from the biobank. In addition, demographic and clinical data of this population are not shown as they could not be recovered from the biobank.

Eighty serum samples from adult patients diagnosed and hospitalised with COVID-19 were obtained during the hospitalisation stay after 20 ± 3 days of the diagnosis to determine the IgG Abs anti-rS and -rN. Based on their signs and symptoms, this population was classified into mild, moderate, and severe COVID-19. Signs and symptoms characterised mild COVID-19 as fever, cough, headache, while a computerised tomography (CT) scan revealed 1–5 points per affected lobe. Moderate COVID-19 was characterised by clinical signs and symptoms of influenza-like disease, including rhinorrhoea, muscle or joint pain, conjunctival hyperaemia, sore throat, and CT revealed > 5–15 points per affected lobe. In addition to the signs/symptoms described above, severe COVID-19 included respiratory distress, intensive care unit attention, and > 15 points per affected lobes in CT. The main characteristics of the hospitalised patient cohort are described in Supplementary Table 1.

Since HIMFG was a reference hospital for COVID-19 attention during the pandemic, 40 samples were collected from the convalescent population 30 ± 3 days after presenting signs and symptoms of COVID-19 to determine the specific IgG Ab. The general characteristics of the convalescent paediatric population are described in Supplementary Table 2. The viral load was defined in this population as described by Flores-Alanis et al. [22]. It was calculated using cycle threshold (Ct) values from RT‐qPCR outputs, using the following equation: y = − 3.2733x + 38.59. Low viral load was defined as Ct value 30–38 (4.2 × 10^2^–1.5 copies number/mL), moderate as Ct value 25–29 (1.4 × 10^4^–8.5 × 10^2^ copies number/mL), and high as Ct value < 24 (< 2.8 × 10^4^ copies number/mL).

In Mexico, COVID-19 vaccination began in December 2020, with the administered formulations based on messenger RNA (BNT162b2 and mRNA-1273) and non-replicating viral vector (ChAdOx1 nCoV-19, Adv5-nCoV and Sputnik V). From these, Adv5-nCoV was required at a single dose, whereas ChAdOx1 nCoV-19, Sputnik V, BNT162b2 and mRNA-1273 required the application of two doses to generate a more robust immune protection. Therefore, the vaccine boost was administered in all populations one year later. This boost was applied either in a homologous or heterologous vaccination strategy, depending on the type of available vaccine. The sampling of whole blood in COVID-19-vaccinated volunteers was obtained as follows: (1) Basal sample (M0) before the first dose; (2) first dose-representing sample (M1) 21‒30 days (only Adv5-nCoV) or before the second dose; (3) second dose-representing sample (M2) ~ 30 days later (only BNT162b2, ChAdOx1 nCoV-19 and Sputnik V); (4) long-lasting evaluation (M3) was sampled six months after first dose administration; and (5) Sample of boost dose (M4) was obtained one year after first dose administration. The main characteristics of the COVID-19 vaccinated volunteers are described in Supplementary Table 3.

### Recombinant S and N protein purification

The rS and rN proteins from SARS-CoV-2 were expressed in the human cell line HEK293T or *E. coli*, respectively. Both recombinant proteins were purified by immobilised metal affinity chromatography (IMAC; Ni-NTA) in native conditions, according to Cortés-Sarabia et al. [23]. The Wuhan SARS-CoV-2 S protein contains six prolines substituted, a substitution of “GSAS” (furin cleavage site), and the addition of a C-terminal fold on the trimerisation motif with an eight-histidine tag that generates a prefusion-stabilised rS protein. Briefly, rS protein (stabilised pre-fusion trimer) was expressed in HEK293F (Thermo Scientific cat# R79007) using SHexaPro plasmid (Addgene cat#: 154754), cells were cultured in FreeStyle^tm^ 293 expression medium (Gibco cat# K900001) at 37 °C and 5% CO_2_, for 48 h. The supernatant was centrifuged and filtered to concentrate the proteins. The IMAC in native conditions was used to purify the rS protein that contains the 8X His tag.

The sequence encoding the Wuhan SARS-CoV-2 N protein (full length) was amplified from RT-PCR using total RNA purified from the swab sample of a child with SARS-CoV-2 infection (2020). By aligning the sequence obtained by Sanger sequencing, it was confirmed that the N sequence belonged to the reference SARS-CoV-2 strain (Wuhan). Following established protocol, the amplified gene was cloned in pLATE 51 (Thermo Scientific cat# K1261). The plasmid pLATE-N was then transformed into Rosetta™(DE3) *E. coli* strain (Sigma-Aldrich cat# 70954) and purified in native conditions by IMAC.

### Indirect enzyme-linked immunosorbent assay (ELISA)

The IgG Abs against the rS and rN proteins from SARS-CoV-2 were determined using the validated in-house method of indirect enzyme-linked immunosorbent assay (ELISA) described in Cortés-Sarabia et al. and Flores-Alanis et al. [22, 23]. Note that our in-house ELISA method was validated through a comparison with a commercial method; from this comparison, a Spearman correlation of 0.8 was obtained. For S protein, the SARS-CoV-2 Spike IgG ELISA Kit (Enzo, ENZ-KIT190) was used, whereas for N protein, the SARS-CoV-2 Nucleocapsid IgG ELISA Kit (Enzo, ENZ-KIT193) was used. Briefly, 96-well plates (Corning cat# 336) were coated with 20 ng/well for rS protein and 10 ng/well for rN protein in 100 µL 0.2 M sodium carbonate-bicarbonate buffer pH 9.4 (Thermo Scientific cat#AAJ67013AE), incubated at 37 °C for 1 h and washed 5 min three times with phosphate-buffered saline (PBS)-Tween 20 (T; 0.05%). Plates were blocked with PBS-T with 5% skimmed milk (Difco cat# 232100) in 200 µL/well, incubated at 37 °C for 40 min, and washed for 5 min three times. Serum samples (1:25 dilution), in triplicate in 100 µL/well, were incubated at 37 °C for 1 h and washed for 7 min three times. Later, 100 µL/well mouse monoclonal anti-human IgG coupled to horseradish peroxidase (Sigma-Aldrich cat# AP112P) was added in 1:2,000 dilution, incubated at 37 °C for 1 h, and washed for 7 min five times. The enzymatic reaction was developed using *o*-phenylenediamine dihydrochloride (Sigma-Aldrich cat# P1526) and stopped using 2 N H_2_SO_4_ (J.T. Baker cat# 9681-02). The resulting optical density (OD) was measured at 492 nm using a microplate reader (Thermo Scientific).

Since storage at room temperature for several weeks and repeated freeze–thaw cycles can increase the level of optical density in sera used for determining either antibodies or analytes [24, 25], it was sought that samples from each condition had a negative control with similar conditions. Thus, for evaluation of Abs from hospitalised patients (severity of the disease) and the convalescent children (viral load), serum samples collected from 30 SARS-CoV-2-positive patients (confirmed by RT-qPCR) were used as positive controls, with at least 15 days after the end of symptoms. In contrast, samples collected from 30 SARS-CoV-2-negative patients (with a negative RT-qPCR result) were used as negative controls, depending on the case of adults or paediatrics. On the other hand, in the cross-reactivity study, 30 sera from a pre-pandemic collection (2018–2019) were used as negative controls. In each group, the cut-off value was calculated following the statistical method described by Frey et al.. (1998) [26]. The equation $\:Cutoff=\stackrel{-}{x}+SD\:t\sqrt{1+(1/n)}$) was used, where X is the mean of independent negative control sera readings, SD is the standard deviation, *n* is the number of independent controls, *t* is the (1-α) percentile of the one-tailed *t*-distribution with υ = *n* – 1 degrees of freedom. Note that in all readings, the background was subtracted (OD = 0.06). Thus, samples with OD >0.37 (cutoff value) were considered positive in determining cross-reaction Abs. On the other hand, a cut-off value of OD >0.2 was considered positive in the evaluation of convalescent children and hospitalised patients. At the same time, in the vaccinated population, the cutoff was OD >0.22.

### Statistical analysis

IgG Ab prevalence was reported as a seropositivity rate (SR) for serum samples obtained before and during the COVID-19 pandemic. The reactivity intensity (RI) value was considered the OD and was reported as the median in serum samples obtained during the COVID-19 pandemic. RI in serum samples obtained from vaccinated healthy volunteers was also reported as the median.

The non-parametric Mann-Whitney U test was conducted between RI and viral load, and the non-parametric Kruskal-Wallis test was conducted between RI and the dose/booster, previous disease, and type of vaccine in the COVID-19-vaccinated population. Contingency table and *Chi*-square test were used to analyse the relationship between categorical variables in the dataset. *p* < 0.05 was considered statistically significant. All statistical analyses were performed using RStudio version 3.2.2, and the graphic representation was conducted using GraphPad Prism version 9.0.0 for Windows (GraphPad Software, San Diego, California, USA; www.graphpad.com).

The identity matrix from the reference sequence of hCoVs S and N proteins was created on Clustal Omega (https://www.ebi.ac.uk/jdispatcher/msa/clustalo) using the following ID from UniProt (https://www.uniprot.org/): hCoV-NL63 S: Q6Q1S2, N: Q6Q1R8; hCoV-229E S: P15423, N: P15130; hCoV-HKU1 S: Q0ZME7, N: Q5MQC6; hCoV-OC43 S: P36334, N: P33469; MERS-CoV S: K9N5Q8, N: K9N4V7; SARS-CoV-1 S: P59594, N: P59595; and SARS-CoV-2 S: P0DTC2, N: P0DTC9.

## Results

As a strategy for evaluating anti-SARS-CoV-2 IgG Abs, the recombinant (r)N and rS proteins were purified and used as antigens for ELISA. This method determined the prevalence and RI of IgG Abs in serum samples obtained before (2003, 2009, 2011, 2016, and 2017) and during the COVID-19 pandemic (convalescent, severely ill, and vaccinated against COVID-19) to better understand the immune response in the Mexican population before and during the COVID-19 pandemic.

### Cross-reactive antibody against rS and rN proteins from SARS-CoV-2 in serum samples obtained before the COVID-19 pandemic

**Fig. 1:**
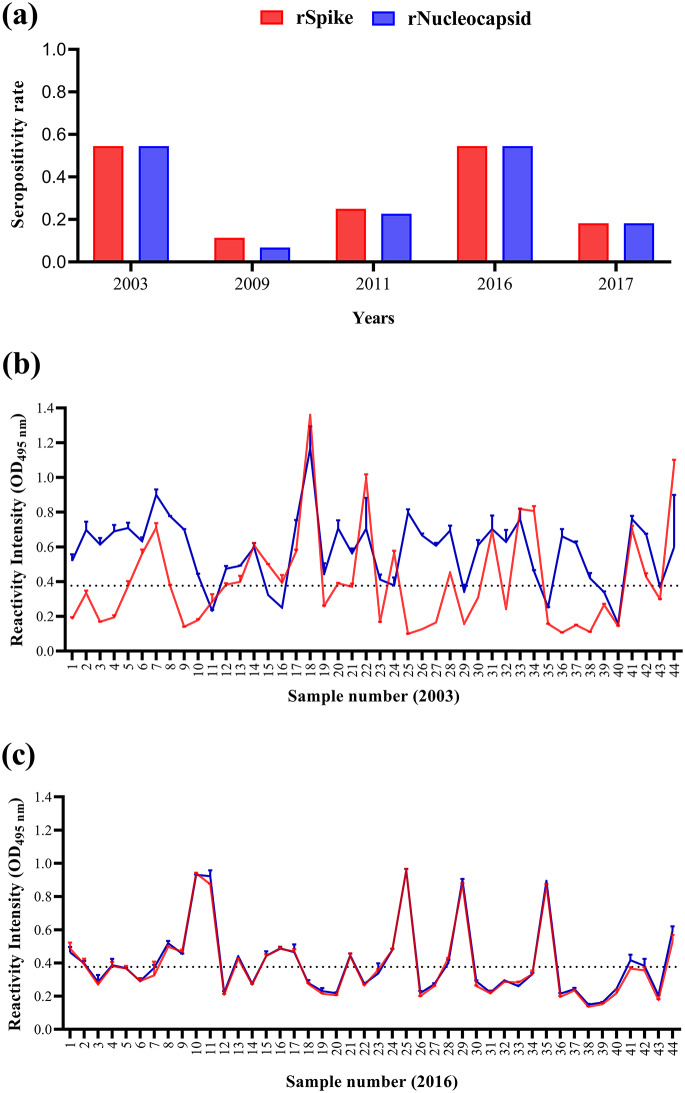
Seropositivity and reactivity intensity of cross-reactive Abs against rS and rN proteins from SARS-CoV-2 in samples obtained before the COVID-19 pandemic. Serum samples (*N* = 220) were sorted according to the collection year into 44 samples in each 2003, 2009, 2011, 2016, and 2017 subgroup year and assessed against recombinant Spike (rS; red) and Nucleocapsid (rN; blue) proteins from SARS-CoV-2 by ELISA. (**a**) Graph showing the seropositivity rate of every evaluated year for both proteins in each serum sample. Graphs showing the O.D. values at 495 nm for the reactivity intensity of rS and rN proteins in 2003 (**b**) and 2016 (**c**) for the 44 sera samples tested. The cut-off value was OD > 0.37

From the 220 serum samples obtained before the COVID-19 pandemic, sorted into five subgroups (2003, 2009, 2011, 2016, and 2017), the highest SR against both rS and rN proteins was observed in the 2003 and 2016 years with 54.4% (24/44 samples) for both years (Fig. 1a). In contrast, the year 2011 showed an SR of 25% (*n* = 11 samples) for rS and 22.7% (*n* = 10 samples) for rN proteins, followed by 2017 with an SR of 18.2% (*n* = 8 samples) for both rS and rN proteins. Finally, 2009 had the lowest SR of all groups, with 11.4% (*n* = 5 samples) for rS and 6.8% (*n* = 3 samples) for rN protein (Supplementary Fig. 2).

Notably, although sera samples from 2003 to 2016 showed the highest SR against rS and rN, those from 2003 showed a differential pattern between rS and rN, with some patients showing a higher RI value for rN, but a low RI for rS (even lower than the cut-off value, Fig. 1b). In contrast, in 2016, the RI value was the same for both proteins (Fig. 1c).

Since the epidemiological data regarding circulating hCoVs in Mexico is scarce for the years of higher RI of rS and rN proteins, a protein sequence alignment from hCoVs was performed to determine percentage values of identity and thus make some inferences about the possible identities of circulating hCoVs that could account for the observed cross-reactivity in 2003 and 2016 (Supplementary Fig. 3). Therefore, the hCoV with the highest identity value for the N protein corresponds to SARS-CoV-1 (89% identity) and MERS-CoV (48% identity) (Supplementary Fig. 3, right panel). Regarding S protein identity, SARS-CoV-1, MERS-CoV, and hCoV-OC43 showed the highest identity values (77%, 31% and 31% of identity, respectively) (Supplementary Fig. 3, left panel). These data suggest that seropositivity in 2016 may be due to SARS-CoV-1, hCoV-NL63, hCoV-229E and hCoV-KU1, with SARS-CoV-1 the most probable. Despite the identity values suggesting that SARS-CoV-1 may account for the seropositivity observed in 2003, this is unlikely because of the year of its emergence (2002), with hCoV-OC43 being the most likely in this year. Thus, further experimental data will be necessary.

### The presence of abs anti-rS and -rN proteins is related to the severity of COVID-19

**Fig. 2:**
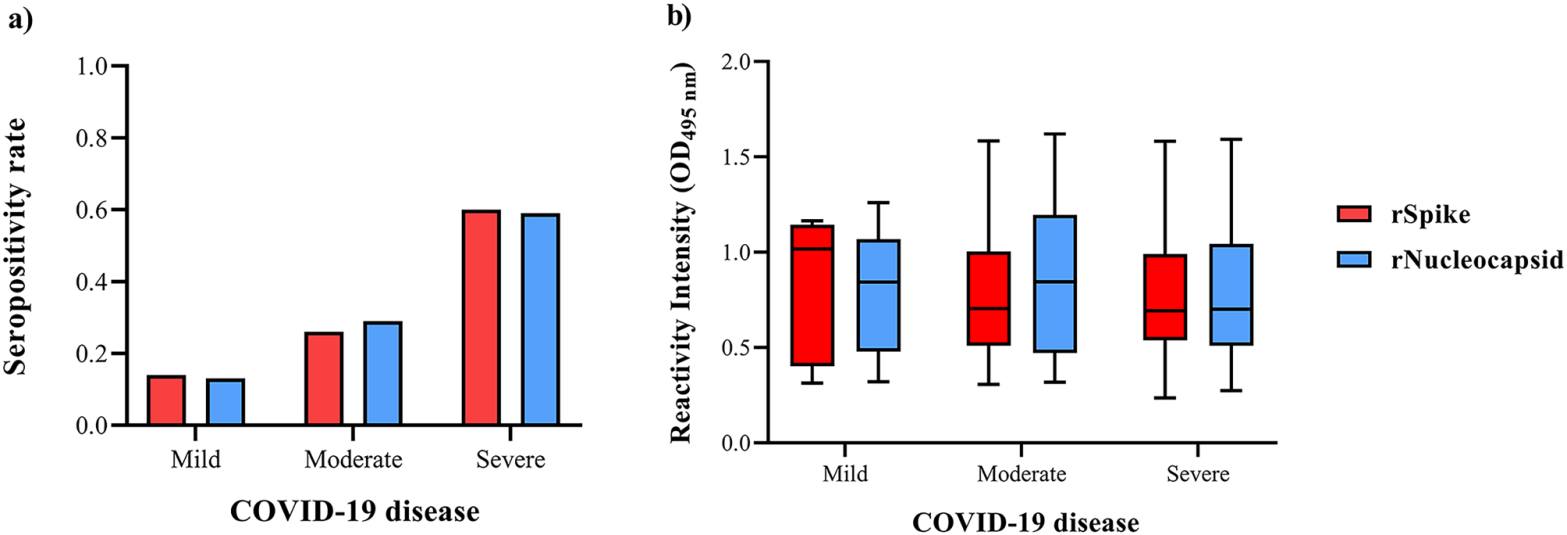
Antibodies anti-rS and -rN proteins from SARS-CoV-2 in mild, moderate, and severe COVID-19. Sera from hospitalised patients (*N* = 80) were obtained during the hospitalisation stay after 20 ± 3 days of the diagnosis. COVID-19 severity was classified according to signs and symptoms as mild (*n* = 17), moderate (*n* = 20), and severe (*n* = 43). Antibodies (Abs) anti-rS (red) and -rN (blue) proteins from SARS-CoV-2 from the obtained sera were evaluated using ELISA. The cut-off value for this cohort was OD > 0.2. (**a**) Seropositivity rate of Abs anti-rS and -rN proteins from SARS-CoV-2 in mild, moderate, and severe COVID-19. (**b**) Reactivity Intensity of Abs anti-rS and -rN proteins (median and interquartile) according to the classification of COVID-19 severity

Of the hospitalised patients for COVID-19, 48.7% were women, and 51.3% were men. The mean age of this population was 59.8 ± 10.8, and 3.7% had mild symptoms, 68.7% moderate, and 27.6% severe. Visual Multilobar Involvement in COVID-19 (VMIC) was used to establish a lung damage score, using the CT. VMIC was classified as having a score of 1 to 6 (Supplementary Table 1). The highest VMIC was identified in 5 score (21 adult patients), which included extensive multilobar involvement (4–5 lobes), clear and prominent central distribution (perihilar, peribronchovascular). Based on the classification of COVID-19 severity, severe disease was the most predominant among the analysed adult patients, with 53.7% (*n* = 43) (Supplementary Table 1). In fact, severe COVID-19 had the highest seropositivity rate when determining Abs anti-rS (60%) and rN (59%) proteins (Fig. 2a). Nevertheless, no differences in SR were observed between Abs anti-rS and -rN proteins among groups (Fig. 2b). According to the population’s characteristics, only age (*p* = 0.012) showed a positive correlation between older patients (68.8 ± 9.7) and COVID-19 severity (Supplementary Table 1).

### IgG Ab response is dependent on SARS-CoV-2 viral load

**Fig. 3:**
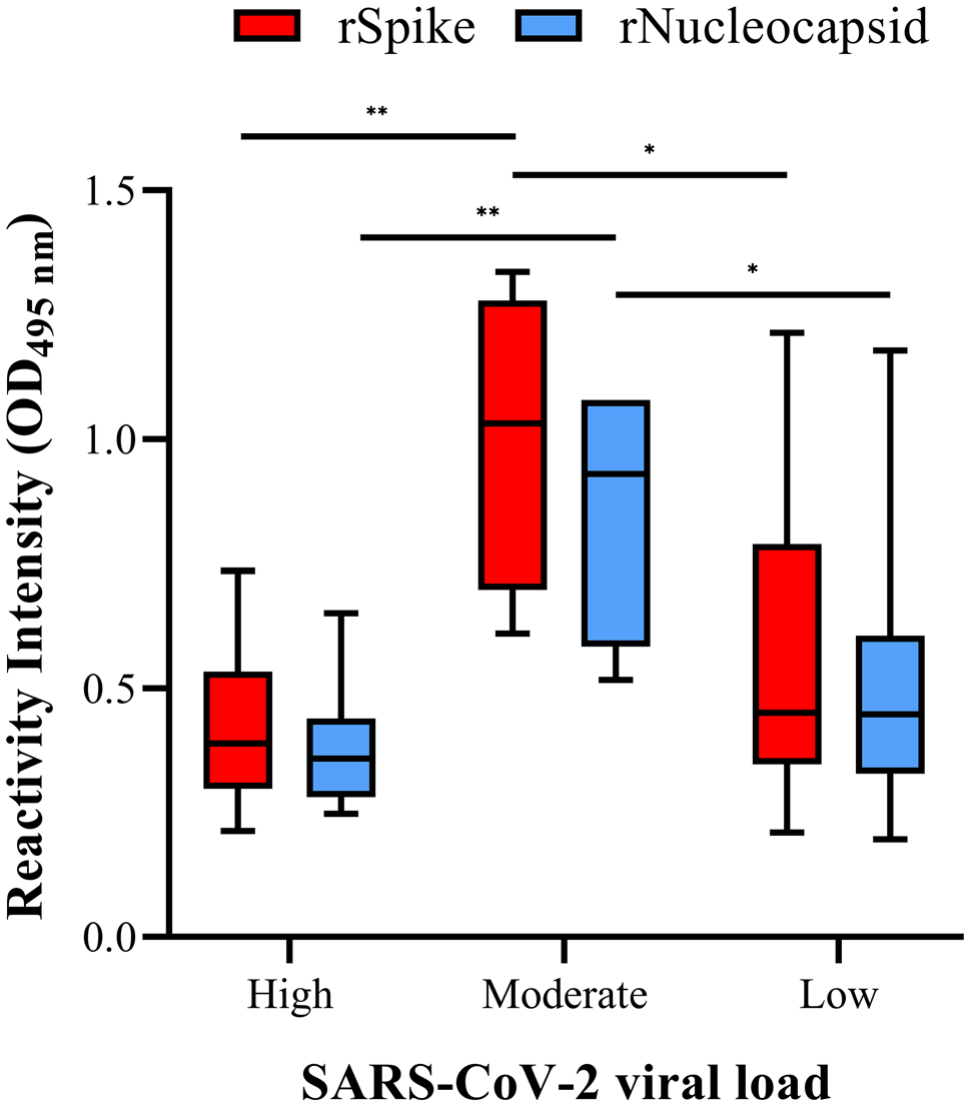
IgG Ab detection (reactivity intensity) against rS and rN proteins from SARS-CoV-2 in convalescent children grouped according to SARS-CoV-2 viral load. Sera from convalescent children (*N* = 40) was obtained 30 ± 3 days after presenting signs and symptoms of COVID-19 and grouped according to their viral load based on the RT-qPCR results (Low viral load was defined as Ct value 30–38, moderate as Ct value 25–29, and high as Ct value < 24). Sera were assessed against Spike (rS; red) and Nucleocapsid (rN; blue) proteins from SARS-CoV-2 by ELISA. The cut-off value was OD > 0.2. Bars represent reactivity intensity (median and interquartile) for rS or rN according to the corresponding SARS-CoV-2 viral load. *p = 0.05, **p ≤ 0.01, ***p ≤ 0.001

The viral load of 40 COVID-19 convalescent paediatric patients aged 0 to 18 years, including 52.5% males (*n* = 21) and 47.5% females (*n* = 19), was calculated. Most of the paediatric patients (57.5%; *n* = 23/40) showed a low viral load, whereas 27.5% of this population presented a high viral load (*n* = 11/40), and only 15% (*n* = 6/40) showed a moderate viral load (Supplementary Table 2). The moderate viral load group showed the highest RI value for anti-rS protein with a median of 1.03 (min 0.6, max 1.33) and anti-rN proteins with a median of 0.93 (min 0.51, max 1.07). When comparing the viral load groups, the moderate group differed from the high group for anti-rS (*p* ≤ 0.01) and anti-rN proteins (*p* ≤ 0.05). In contrast, only the moderate and low groups for anti-rN proteins differed (*p* ≤ 0.05) (Fig. 3).

When characteristics of the paediatric population were analysed, a negative correlation between female paediatric patients and viral load was observed (Supplementary Table 2). Regarding paediatric ages, when they were ranked into infants (younger than 2 years), children (2–9 years) and adolescents (10–19 years), adolescents concentrated most of the convalescent paediatric population (52.5%; *n* = 21/40), but no correlation with viral load was observed. To note, low viral load was higher in the population without comorbidities (42.5%; *n* = 17/40) (Supplementary Table 2).

### SARS-CoV-2 natural infection increases the response of IgG antibody anti-rS in the COVID-19-vaccinated population

**Fig. 4:**
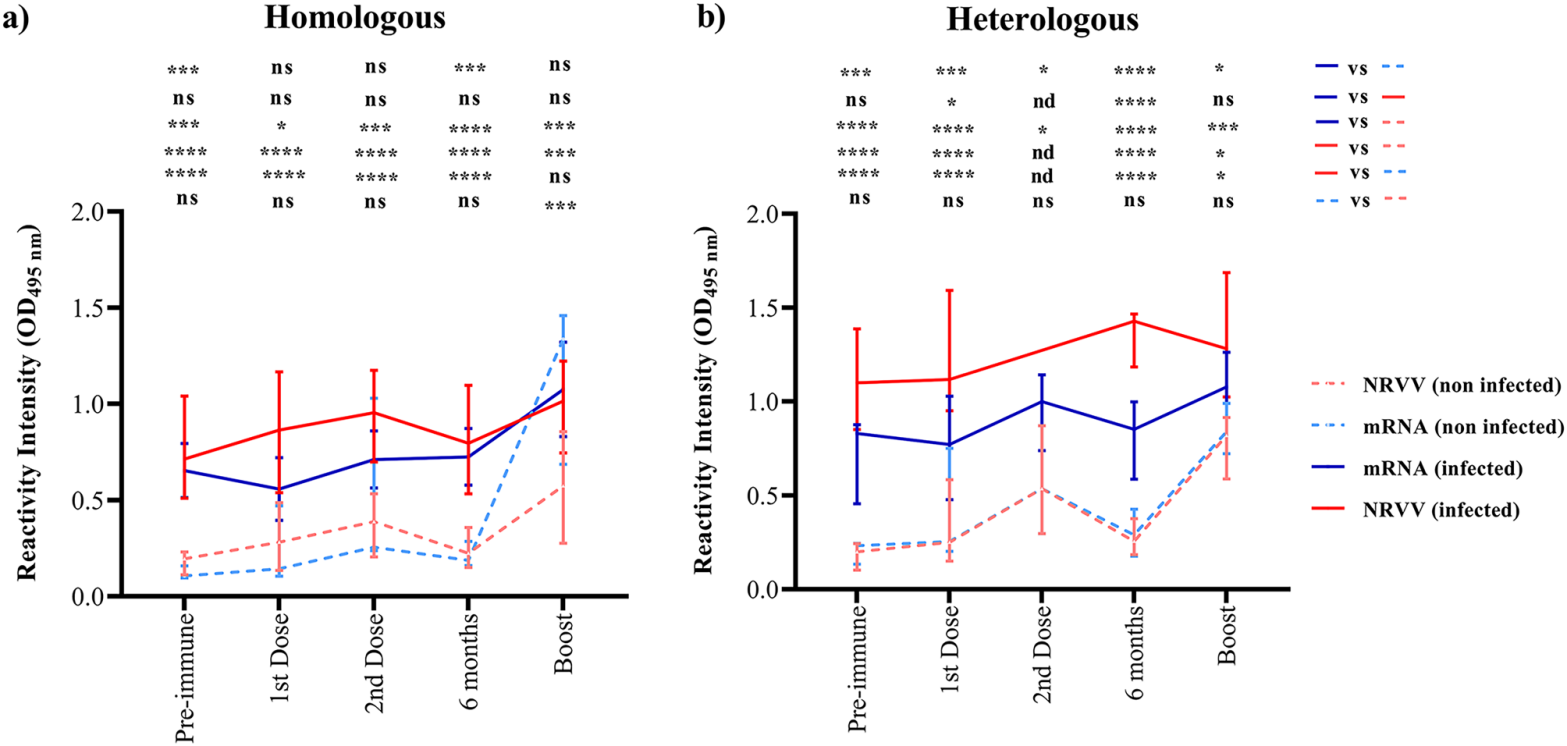
Natural SARS-CoV-2 infection increases reactivity intensity against rS in the COVID-19-vaccinated population. A total of 435 serum samples corresponding to 90 COVID-19-vaccinated volunteers were grouped depending on the immunological state into pre-immune, 1st dose, 2nd dose, 6 months (lasting), and boost. An additional sorting into infected or non-infected was conducted depending on the reactivity intensity against rN. According to the type of vaccine and the scheme of vaccination, the cohort was divided into mRNA vaccine, Non-replicant viral vector (NRVV), homologous (**a**) or heterologous (**b**) vaccination regimen. A Mann-Whitney U test was performed to compare infected vs. non-infected volunteers at every immunological state. The cut-off value was OD > 0.22. *p = 0.05, **p ≤ 0.01, ***p ≤ 0.001. ns = non-significant and nd = not determined

Four hundred and thirty-five serum samples were obtained from 90 COVID-19-vaccinated volunteers aged 25 to 72, of whom 68.8% (*n* = 62) were female and 31.2% (*n* = 28) were male (Supplementary Table 3). Since most vaccines against COVID-19 are based on rS protein as an antigen, anti-rN Abs were analysed in this population to determine a previous natural SARS-CoV-2 infection, in which 77.7% (*n* = 70) had a RI value to rN protein, and 22.3% (*n* = 20) were negative. Notably, the volunteers with pre-infection showed the highest RI value (*p* < 0.0001) for anti-rS with a median of 0.88 (min. 0.24, max. 1.81) when compared with non-infected volunteers with a median of 0.25 (min. 0.09, max. 1.45) (Fig. 4).

Vaccinated volunteers were grouped according to both the type of vaccine platform (mRNA [BNT162b2 and mRNA-1273]) or non-replicating viral vector (NRVV [ChAdOx1 nCoV-19, Sputnik V, and Adv5-nCoV])) and the regimen (homologous or heterologous) that was administered (Fig. 4).

In the first dose, the sera of vaccinated volunteers with NRVV showed differences in RI value against anti-rS Abs with and without SARS-CoV-2 pre-infection (*p* < 0.001) (Fig. 4a). In contrast, in the heterologous regimen, differences in RI value were observed for either NRVV and mRNA vaccinated sera with and without SARS-CoV-2 pre-infection (*p* < 0.001) (Fig. 4a). In contrast, only differences were observed in the sera of vaccinated volunteers with the NRVV homologous regimen in the second dose. To note, six months after the first dose, sera of all vaccinated volunteers had differences in RI value against anti-rS Ab between infected and non-infected with SARS-CoV-2 (*p* ≤ 0.05) (Fig. 4a and b). Interestingly, in the boost administration with the homologous mRNA regimen, a considerable increase in RI value was observed in the non-infected with SARS-CoV-2 group, reaching similar RI values to the infected with SARS-CoV-2 group. Notably, in the boost administration with NRVV in both homologous and heterologous regimens, differences were observed between infected and uninfected volunteers (*p* ≤ 0.05) (Fig. 4a and b).

## Discussion

To date, seven hCoVs have been identified: hCoV-NL63, hCoV-229E, hCoV-HKU1, hCoV-OC43, SARS-CoV-1, MERS-CoV and the responsible for the COVID-19 pandemics SARS-CoV-2 [27, 28]. Since most of these viruses produce severe respiratory disease in humans, much interest has been gained in their study [28]. Despite the high identity among hCoVs, the receptor required for human infection among each hCoV differs. Such differential receptor requirement accounted for the variability of the S1 subunit in length and amino acid sequence of the S protein, as the S2 subunits share a high identity (≥ 50%) [29]. In addition to the S2 subunit, the N protein possesses a higher conservation identity (90%) and few mutations over time [30]. Therefore, an antigenic cross-protection for hCoVs belonging to the same genetic groups has been described to correlate with disease severity in COVID-19 patients. For example, reports from the United Kingdom, USA, Sub-Saharan Africa, China, and Italy demonstrated that humoral response against several seasonal hCoVs can cross-react with SARS-CoV-2, leading to the acquisition of protective immunity that could explain the reduction of COVID-19 severity in some people [18, 19, 31, 32]. Cross-reactivities among the Abs against the N protein from SARS-CoV-2 and the N protein from hCoV-229E, hCoV-NL63, and hCoV-OC43 in sera from Sierra Leonean Lassa fever and Ebola survivors obtained before COVID-19 pandemics was found. In contrast, in sera from COVID-19 subjects and healthy blood donors from the United States, only cross-reactivity for hCoV-NL63 and MERS-CoV was observed [21]. Similarly, in sera of pre-pandemic donors from Sassari, Italy, the presence of antibodies against hCoV-NL63 was able to cross-react with the SARS-CoV-2 epitopes S_421–434_ (FSQILPDPSKPSKRSFIE) and S_742–759_ (CNGVEGFNCYFPLQS) from the spike protein [32]. Overall, these works indicate that previous exposure to hCoVs may produce antibodies that could confer protective immunity against SARS-CoV-2 [32, 33]. In this study, the pre-pandemic sera from 2003 to 2016 showed the highest prevalence of IgG Ab against rS- and rN-SARS-CoV-2. Even though the identity of the hCoVs that generated such Abs was not experimentally determined, it must correlate with the circulating hCoV in Mexico. In Mexico, it was not until 2013 that the Instituto de Diagnóstico y Referencia Epidemiológicos implemented a differential diagnosis of influenza that also included 14 other respiratory viruses (http://www.indre.salud.gob.mx), delimitating the epidemiological information of the circulating hCoVs identity before 2013. A work analysing sera from Mexican subjects aged >3 months presenting an Influenza-like illness from April 2010 to April 2011 showed the presence of hCoV-OC43, hCoV-NL63 and hCoV-229E [34]. A former study in the USA performed between 2003 and 2004 in sera from < 20 years old showed a 34.8–62.5% seropositive rate against hCoV-229e and a 25.0–70.3% seropositive rate against HCoV-NL63 [35]. hCoV-NL63, hCoV-229E and even hCoV-OC43 could likely have been our country’s circulating hCoVs in 2003 and thus be responsible for the higher cross-reactivity this year. Regarding 2016, identity values data suggest that the hCoVs accounting for the cross-reactivity could be SARS-CoV-1, hCoV-NL63, hCoV-229e and hCoV-KU1. In agreement with our work, a report analysing samples of the age groups 0–9 and 10–19 years from the Laboratorio Central de Epidemiología of the Instituto Mexicano del Seguro Social from 2014 to 2015 found that the predominant hCoVs were hCoV-HKU1, hCoV-NL63, hCoV-229E and βCoV1 [36]. Similarly, in another cross-sectional, prospective study from March 2010 to August 2013 using samples of patients aged 1 month to 5 years with pneumonia diagnosis, the predominant hCoVs were hCoV-NL63 (0.8%), hCoV-OC43 (0.6%), hCoV-229E (0.3%) and hCoV-HKU1 (0.1%) [37]. In line with these data, a follow-up study from the National Respiratory and Enteric Virus Surveillance System by U.S. laboratories from July 2014 to June 2017 found that the most common HCoV strains detected were hCoV-OC43 (40%), hCoV-NL63 (20%), hCoV-HKU1 (19%) and hCoV-229E (14%). Interestingly, hCoV-NL63 and hCoV-HKU1 showed larger peaks in January 2016 [38]. These findings suggest that our hypothesis is viable and that hCoV-NL63, hCoV-229E, hCoV-KU1and, and SARS-CoV-1 could be the viruses responsible for the cross-reactivity observed in 2016. However, further experimental research will be necessary to determine the identity of the hCoVs responsible for the cross-reactivity data. In summary, these data suggest that cross-protection depends on circulating hCoVs in specific regions of each country worldwide.

SARS-CoV-2 infection mainly remains asymptomatic, but older adults develop a severe COVID-19 infection depending on their immune status, a prior COVID-19 infection, vaccination, and the circulating strain [17, 39]. In COVID-19 patients, the primary Abs response to SARS-CoV-2 is against S and N proteins [40, 41]. Therefore, one of the main aims of this work was to evaluate IgG Ab against rS and rN from SARS-CoV-2 in sera of patients with mild, moderate, or severe COVID-19 infection. Patients with severe disease showed the highest seropositivity, followed by moderate and mild COVID-19. The impaired tendency of IgG Ab production anti-SARS-CoV-2 could be due to infection control and ageing. Still, in this work, a correlation between advanced age and COVID-19 severity was observed in the hospitalised adult population, indicating that in this population, age represents a risk factor in COVID-19 severity. Failure of IgG Ab production against SARS-CoV-2 causes uncontrolled viral replication in the airways and activates an unregulated immune response [42]. Interestingly, an association between lung damage and IgG Abs anti-rN protein was observed in this population. SARS-CoV-2 can induce autoimmune and autoinflammatory states along with tissue invasion, and the development of immunosuppressive hyperallergic mechanisms of systemic inflammation is critical at every stage of infection [43]. Moreover, when non-nAbs bind to viral antigen, viral entry is induced, increasing viral infection and replication, even excessive antibody Fc-mediated effector functions or immune complexes could lead to antibody-dependent enhancement (ADE), aggravating COVID-19 outcome [44]. In this sense, macrophages internalise SARS-Cov-2 N protein released from nearby infected cells; this process is assisted by Fc receptor, resulting in cytokine secretion from macrophages, cytokine storm, and severe disease [45]. Non-nAbs or ADE can generate damage and aggravate the disease state and outcome.

Viral infection is a highly complex process in which the interplay between host and viral factors shapes viral replication and shedding dynamics. Variation in viral load among individuals is due to host susceptibility and immunity from previous infection and vaccination [46]. Since viral load can be associated with the propensity to transmit the virus, heterogeneity between individuals has an essential role in ongoing viral transmission [46, 47]. To note, this heterogeneity was observed in the infected population when evaluating the IgG Abs anti-rS and -rN from SARS-CoV-2, in which patients with moderate viral load had the highest RI, probably due to a well-controlled infection. Unregulated circumstances such as high viral load, antibodies and cellularity fail to control infection, leading to the peak of the viral load [48, 49]. On the other hand, a low viral load is easier to eliminate; therefore, a low IgG Ab level with high neutralising activity could be enough to accomplish the goal. Thus, further neutralising experiments will help to get a better understanding of this process.

Several COVID-19 vaccines, deployed as diverse vaccine platforms, successfully protect against SARS-CoV-2, remarkably reducing hospitalisation and death rates [50–57]. Close comparisons of Abs responses to diverse vaccines in humans may help understand protective immunity against COVID-19, with a particular scope in immune memory [58]. Although natural infection induces a stronger and potentially effective immune response, vaccination remains the best strategy to induce an immune response safely [59–64]. In this study, basal sera from volunteers with natural pre-infection showed a remarkable level of anti-rS-SARS-CoV-2 IgG Abs compared to non-infected volunteers. Interestingly, only the sera from prime vaccination with the mRNA vaccines showed a similar level of anti-rS to pre-infected volunteers. In this regard, previous work also demonstrated that in sera from Mexican volunteers, the mRNA BNT162b2 vaccine generated the highest Ab levels in basal sera from the population with SARS-CoV-2 pre-infection [65]. In contrast, sera with the complete vaccination schedule had similar levels with or without pre-infection [66]. On the other hand, sera from a boost with homologous mRNA and heterologous (NRVV) showed identical results. A follow-up study from Mexican and Argentinian volunteers recently described the Abs response to the prime/boost vaccination scheme with a similar combination of vaccines (homologous and heterologous administration) [67]. The results obtained were identical to those found in this study; the homologously administered mRNA vaccine and heterologously administered NRVV boost vaccines were the most effective in inducing higher Abs levels [68]. Similar results have been reported in vaccinated volunteers from Germany and the UK [69, 70], suggesting that mRNA vaccines can induce an efficient immune response comparable to that obtained from SARS-CoV-2 infection. Goel et al. (2021) showed that the BNT162b2 vaccine can induce peak levels of anti-spike IgG Abs compared to mild or moderate natural infection [71]. The combination of prior infection and vaccination yields the most potent and broad neutralisation capacity against variants. Neutralising activity is generally vigorous following BNT162b2 vaccination [72]. However, vaccine-induced Abs tend to have higher avidity early after the second dose due to rapid affinity maturation, while natural infection is due to a more diversified B cell response with broader cross-reactivity [73].

## Conclusions

Epidemiological outbreaks and circulating non-SARS-CoV-2 coronaviruses, mainly hCoV-229E and hCoV-NL63, account for the leading causes of the cross-reaction Abs, which could be implied in the protection against SARS-CoV-2 infection. In addition, IgG Ab levels in SARS-CoV-2 infected patients may be related to disease severity, which could be associated with viral load and age. In the COVID-19-vaccinated population, the levels of IgG Abs anti-rS protein are influenced by previous disease, type of vaccine, and whether vaccine administration was homologous or heterologous. Interestingly, the BNT162b2 vaccine generated the best humoral immune response in the Mexican population. Evaluating the Abs-based humoral immune response in serum samples obtained before and during an outbreak/pandemic could help understand emerging/reemerging diseases and develop efficient preventive strategies.

## Supplementary Information

Below is the link to the electronic supplementary material.

Supplementary Material 1

## Data Availability

The HIMFG data supporting this study’s findings are available from the corresponding author (V.L.) upon reasonable request.

## Abbreviations

r: recombinant
S: Spike
N: Nucleocapsid
Abs: Antibodies
RI: Reactivity intensity
CoVs: Coronaviruses
h: human
SARS: Severe acute respiratory syndrome
MERS: MIDDLE East respiratory syndrome
VOC: Variants of concern
SR: Seropositivity rate
CAT: Computed axial tomography
Ct: Cycle threshold
M0: Basal sample
M1: First dose-representing sample
M2: Second dose-representing sample
M3: Sample of long-lasting evaluation
M4: Sample of boost dose
ELISA: Enzyme-linked immunosorbent assay
PBS: Phosphate-buffered saline
T: Tween 20
OD: Optical density
X: Mean
SD: Standard deviation
