# Supplementary material for: Cross-reactivity IgG, viral load, severity and vaccination outcome as an approach for understanding humoral immune response against SARS-CoV-2
Source: BMC Infect Dis. 2025 Nov 18;25:1606. doi: 10.1186/s12879-025-12038-3 (PMC12625219; doi:10.1186/s12879-025-12038-3)
Supplement: Supplementary file 1 — Supplementary Material 1 [file 12879_2025_12038_MOESM1_ESM.docx]

Cross-reactivity IgG, viral load, severity and vaccination outcome as an approach for understanding humoral immune response against SARS-CoV-2

Jesus Contreras-Villa^1,2^, Griselda Rodríguez-Martínez^1^, Israel Parra-Ortega^3^, Mariana Romo-Castillo^1,4^, Karen Cortés-Sarabia^5^, Zeus Saldaña-Ahuactzi^6^, Alejandro Flores-Alanis^7^, Alfredo Aureoles-Romero^1^, Marcela Salazar-García^8^, James González^9^, Carlos A. Eslava-Campos^10^, Ulises Hernández-Chiñas^10^, Armando Cruz-Rangel^11^, Rosario Morales-Espinosa^7^, Mario Eugenio Cancino-Diaz^2^, and Victor M. Luna-Pineda^1,^*


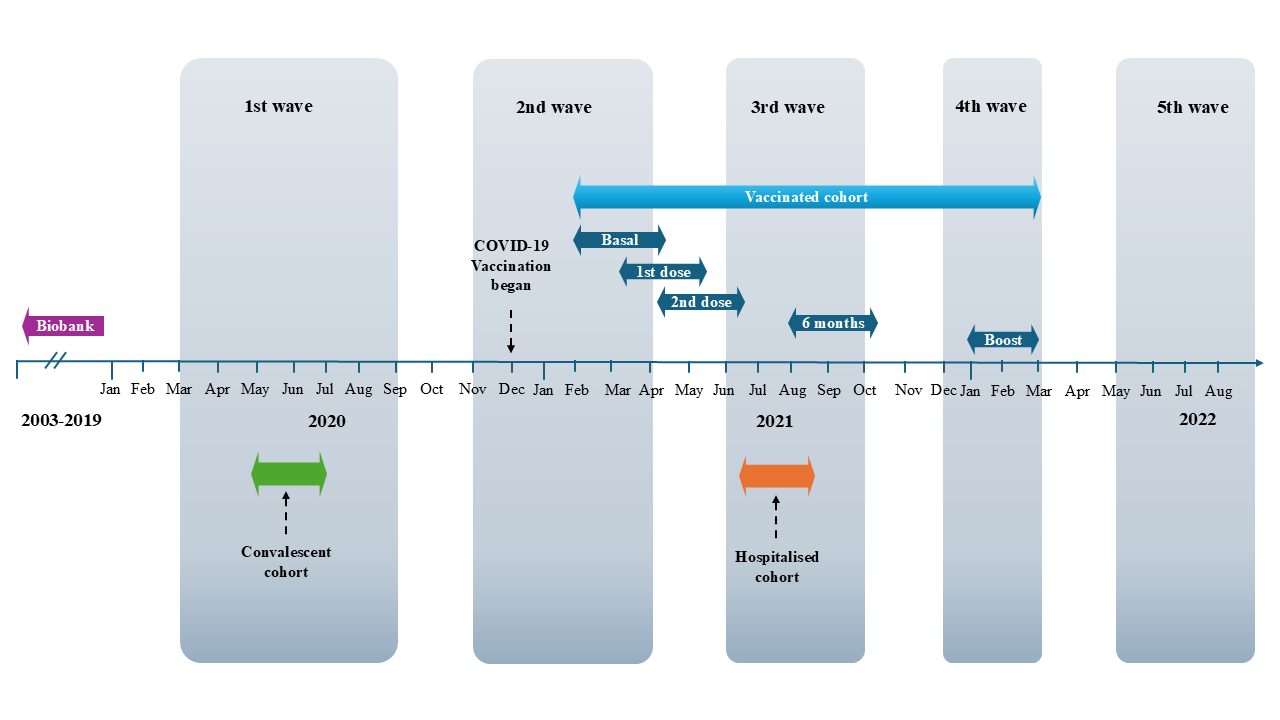


**Supplementary Figure 1. Schematic representation of sample collection times in the several cohorts used in this study**. Samples were collected as follows: Pre-pandemic samples (purple arrow) were obtained from the Biobank sera from 2003 – 2009. Sera from the convalescent cohort (green arrow) were obtained from April to July 2020. Basal samples were obtained in the vaccinated cohort (light blue arrow) before receiving the first vaccine shot. In contrast, samples belonging to the first dose were collected 25 ± 5 days after receiving the first vaccine shot, but previous to the second vaccine shot (Adv5-nCoV has only one dose, lower panel); acquisition of samples from the second dose was 25 ± 5 days after receiving the second vaccine dose. The 6-month-long sample corresponded to samples obtained 175 ± 5 days after receiving the first vaccine shot, and boost samples were obtained 14 ± 3 days after receiving the corresponding vaccine boost dose (BNT162b2, ChAdOx1 nCoV, Sputnik V, mRNA-1273). Hospitalised cohort samples (orange arrow) were acquired from June to September 2021. In all cases, sera were obtained, aliquoted, and stored at -80°C until used.


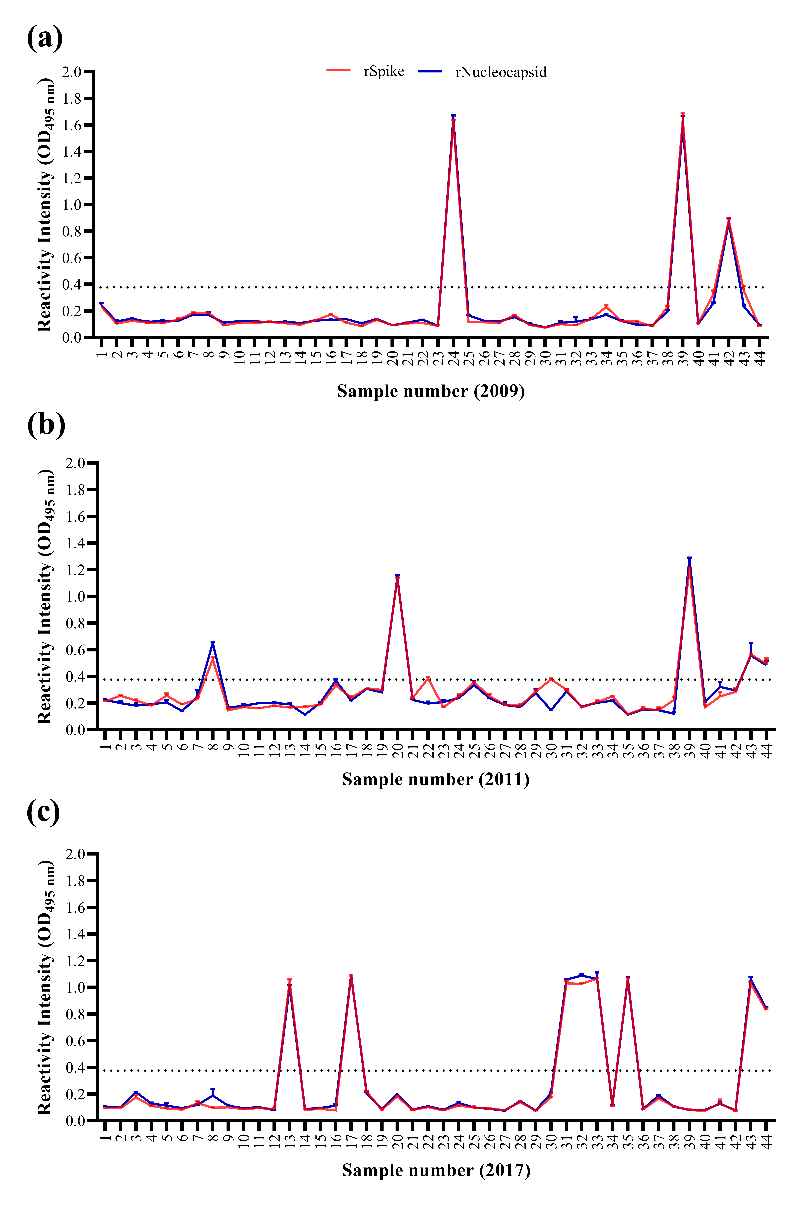


**Supplementary Figure 2. Reactivity intensity of cross-reactive Abs against rSpike and rNucleocapsid proteins from SARS-CoV-2 in samples obtained before the COVID-19 pandemic.** Serum samples from 2009 (a), 2011 (b) and 2017 (c) were assessed against recombinant Spike (rS; red) and Nucleocapsid (rN; blue) proteins from SARS-CoV-2 by ELISA. The cut-off value was OD > 0.37.


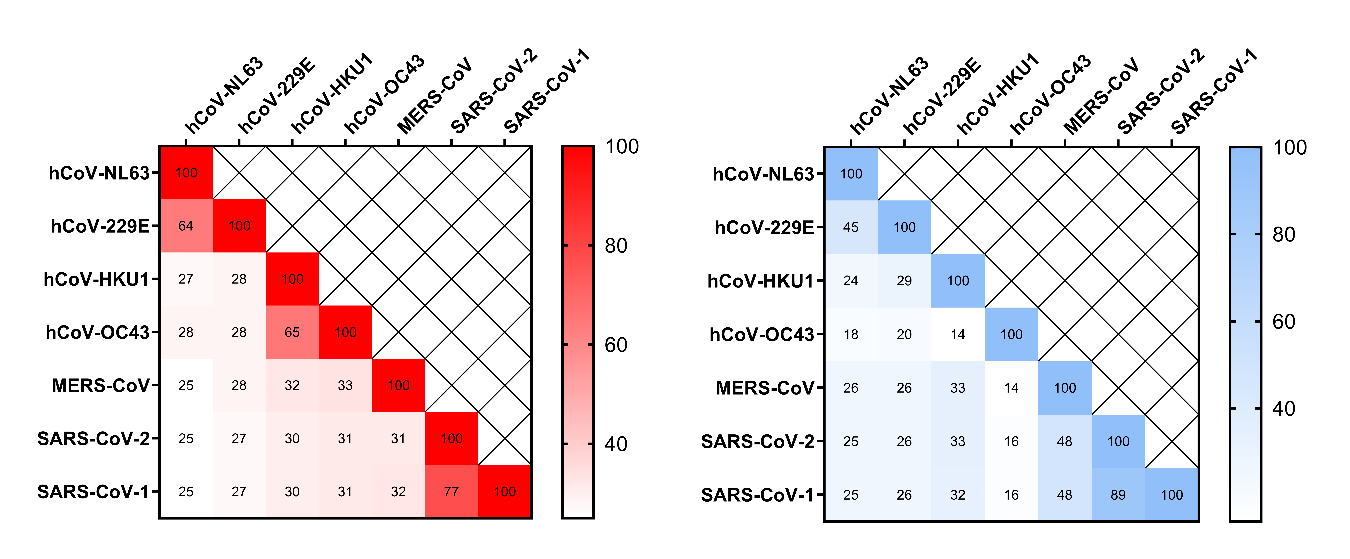


**Supplementary Figure 3. Human Coronavirus (hCoVs) spike (S) and nucleocapsid (N) proteins’ identity**. The heat map on the left panel represents the S's identity at the protein level among several hCoVs. In contrast, the right panel shows the N protein identity among hCoVs. In both cases, S or N protein sequences from hCoVs were aligned on Clustal Omega (https://www.ebi.ac.uk/jdispatcher/msa/clustalo) to determine percentage values of identity.

**Supplementary Table 1. Main characteristics of the hospitalised adult patient cohort.**

| COVID-19 Severity | | | |
| --- | --- | --- | --- |
| Characteristics % (N = 80)* | Mild % (n)^1^ | Moderate % (n)^2^ | Severe % (n)^3^ |
| Sex | | | |
| Male: 51.3 (42) | 14.2 (6) | 23.8 (10) | 62 (26) |
| Female: 48.7 (38) | 29 (11) | 26.3 (10) | 44.7 (17) |
| Age (59.8 ± 10.8) | 55.4 ± 11.2 | 59.9 ± 11.1 | 68.8 ± 9.7 |
|  | Ref. | *p* = 0.3222 | ***p* = 0.0121** |
| Symptoms | | | |
| Mild 3.7 (3) | 100 (3) | 0 (0) | 0 (0) |
| Moderate 68.7 (55) | 25.4 (14) | 36.4 (20) | 38.2 (21) |
| Critical 27.6 (22) | 0 (0) | 0 (0) | 100 (22) |
| VMIC scale | | | |
| 1: 21.2 (17) | 100 (17) | 0 (0) | 0 (0) |
| 2: 6.3 (5) | 0 (0) | 60 (3) | 40(2) |
| 3: 20 (16) | 0 (0) | 93.7 (15) | 6.3 (1) |
| 4: 20 (16) | 0 (0) | 12.5 (2) | 87.5 (14) |
| 5: 26.2 (21) | 0 (0) | 0 (0) | 100 (21) |
| 6: 6.3 (5) | 0 (0) | 0 (0) | 100 (5) |
| 7: 0 (0) | 0 (0) | 0 (0) | 0 (0) |

*Samples collected in 2020 showing the percentage values of the main characteristics of the hospitalised population. Age is represented as the mean value ± SD. *p* values ≤ 0.05 are shown in bold to denote statistical significance.

**^1^**Fever, cough, headache, computerised tomography (CT) scan revealed 1–5 points per affected lobe.

**^2^** Signs/symptoms described in 1, including rhinorrhoea, muscle or joint pain, conjunctival hyperaemia, and sore throat. >5–15 points per affected lobes.

**^3^** Respiratory distress, intensive care unit attention, >15 points per affected lobes.

Mild symptoms: fever, cough, headache

Moderate symptoms (additional): Rhinorrhoea, muscle or joint pain, conjunctival hyperaemia, sore throat.

Critical symptoms (additional): respiratory distress

Ref: reference

VMIC: Visual Multilobar involvement in COVID-19

VMIC 1: Normal CT scan, no lung involvement

VMIC 2: Involvement of 1–2 lobes, only peripheral opacities, no central involvement

VMIC 3: Mild multilobar involvement (≥2 lobes), minimal or doubtful central zone involvement

VMIC 4: Moderate multilobar involvement (3–4 lobes), definite central involvement in some segments

VMIC 5: Extensive multilobar involvement (4–5 lobes), clear and prominent central distribution (perihilar, peribronchovascular)

VMIC 6: Diffuse involvement with both central and peripheral opacities, bilateral, mixed ground-glass and consolidation patterns

VMIC 7: Severe or critical lung damage: diffuse, dense opacities with loss of central–peripheral distinction, ARDS-like pattern

**Supplementary Table 2. Main characteristics of the convalescent paediatric population.**

| SARS-CoV-2 viral load | | | |
| --- | --- | --- | --- |
| Characteristics (N = 40)^*^ | Low % (n)^1^ | Moderate % (n)^2^ | High % (n)^3^ |
| Sex | | | |
| Male: 52.5 (21) | 47.8 (10) | 14.2 (3) | 38 (8) |
|  | Ref. | *p* = 0.052 | *p* = 0.637 |
| Female: 47.5 (19) | 68.6 (13) | 15.7 (3) | 15.7 (3) |
|  | Ref. | ***p* = 0.012** | ***p* = 0.012** |
| Age | | | |
| Infancy: < 2 years: 10 (4) | 75 (3) | 0 (0) | 25 (1) |
|  | Ref. | nd | nd |
| Childhood: 2‒9 years: 37.5 (15) | 53.3 (8) | 13.3 (2) | 33.3 (5) |
|  | Ref. | nd | *p* = 0.405 |
| Adolescence: 10‒19 years: 52.5 (21) | 57.2 (12) | 19 (4) | 23.8 (5) |
|  | Ref. | *p* = 0.405 | *p* = 0.089 |
| Patient care | | | |
| Ambulatory: 57.5 (23) | 56.5 (13) | 17.5 (4) | 26 (6) |
|  | Ref. | ***p* = 0.029** | *p* = 0.108 |
| Hospitalised: 42.5 (17) | 58.8 (10) | 11.8(2) | 29.4 (5) |
|  | Ref. | ***p* = 0.021** | *p* = 0.197 |
| Comorbidities | | | |
| No comorbidities: 70 (28) | 60.7 (17) | 14.2 (4) | 25 (7) |
|  | Ref. | ***p* = 0.004** | ***p* = 0.041** |
| Comorbidities: 30 (12) | 50 (6) | 16.6 (2) | 33.4 (4) |
|  | Ref. | nd | *p* = 0.527 |

* Samples collected in 2020 showing the percentage values of the main characteristics of the convalescent paediatric population. *p* values ≤ 0.05 are shown in bold to denote statistical significance.

**^1^** Ct value corresponds to 30–38 (4.2 × 10^2^–1.5 copies number/mL).

**^2^** Ct value 25–29 (1.4 × 10^4^–8.5 × 10^2^ copies number/mL).

**^3^** Ct value <24 (<2.8 × 10^4^ copies number/mL).

Ref: reference

nd: not determined

**Supplementary Table 3.** **The main characteristics of the COVID-19 vaccinated volunteers.**

| **Cohort (ref.)** | **Gender** | **Previous SARS-CoV-2 infection status** | **COVID-19 vaccine (Prime-Boost)** |
| --- | --- | --- | --- |
| **COVID-19 vaccinated volunteers**  **N = 90 ***  **Aged 25-72** | Female 68.8%  (n = 62) | Infected 77% (n =70) | BNT162b2-BNT162b2 5.5% (n = 5) |
|  |  |  | BNT162b2-ChAdOx1 nCoV-19 14.4% (n = 13) |
|  |  |  | BNT162b2-Sputnik V 1.1% (n = 1) |
|  |  |  | ChAdOx1 nCoV-19-ChAdOx1 nCoV-19 34.4% (n = 31) |
|  |  |  | ChAdOx1 nCoV-19-Sputnik V 8.8% (n = 8) |
|  | Male 31.2% (n = 28) | Non-infected 23% (n = 20) | ChAdOx1 nCoV-19-mRNA-1273 1.1% (n = 1) |
|  |  |  | Sputnik V-ChAdOx1 nCoV-19 12.2% (n = 11) |
|  |  |  | Sputnik V-Sputnik V 4.44% (n = 4) |
|  |  |  | Adv5-nCoV-Sputnik V 2.2% (n = 2) |
|  |  |  | Adv5-nCoV-ChAdOx1 nCoV-19 6.6% (n = 6) |
|  |  |  | Adv5-nCoV-mRNA-1273 7.7% (n = 7) |

* Samples collected between 2021-2022
